# Incorporation of lipids improves cryo-tolerance of vitrified gorgonian coral oocytes

**DOI:** 10.1371/journal.pone.0341229

**Published:** 2026-02-19

**Authors:** Yen-Po Chen, Tzu-Fei Huang, Ester Lo, Sujune Tsai, Zhi-Hong Wen, Hung-Kai Chen, Chiahsin Lin

**Affiliations:** 1 Department of Obstetrics and Gynecology, Kaohsiung Armed Forces General Hospital, Kaohsiung, Taiwan; 2 Department of Obstetrics and Gynecology, Tri-Service General Hospital, National Defense Medical Center, Taipei, Taiwan; 3 National Museum of Marine Biology and Aquarium, Pingtung, Taiwan; 4 Department of Marine Biotechnology and Resources, National Sun Yat-sen University, Kaohsiung, Taiwan; 5 Graduate Institute of Marine Biology, National Dong Hwa University, Pingtung, Taiwan; Friedrich-Loeffler-Institute, GERMANY

## Abstract

Coral reefs, which are crucial to the health and diversity of the global marine ecosystem, have experienced substantial declines due to climate change and human activities. Cryopreservation, the long-term storage of biological material at ultra-low temperatures, represents a novel strategy for coral conservation. The current study investigated the effects of integrating specific liposomes into vitrification solutions (VS) on the viability of oocytes from two coral species, oocytes were vitrified using a VS containing one of eight lipids. The study results indicate that incorporating PE and erucic acid considerably enhances the viability of *J. juncea* oocytes, whereas incorporating PC reduces their viability. However, incorporating palmitoleic acid, docosahexaenoic acid, linoleic acid, and eicosatrienoic acid did not substantially affect the viability of *J. juncea* oocytes. In *J. fragilis*, erucic acid yielded the highest viability (54.12% ± 2.99%), with oleic acid and docosahexaenoic acid also showing positive effects. Among the tested liposomes, erucic acid led to the highest overall viability in both species. Lipid-enhanced vitrification can assist coral conservation by facilitating cryobanking of coral gametes and supporting their postthaw development, which can assist with coral reef restoration.

## Introduction

Coral reefs, which are crucial to the health and diversity of the global marine ecosystem, have experienced a considerable decline over the past 2 decades due to human activities and climate change. According to the Status of Coral Reefs of the World: 2020 report, approximately 14% of global coral reefs were lost between 2009 and 2018 due to rising ocean temperatures, acidification, and pollution. [1]. Frequent coral bleaching events, driven by prolonged increasing sea temperatures, have had devastating effects on reef ecosystems [2]. Mass coral bleaching episodes in 1998, 2010, and 2015 collectively impacted over 70% of coral reefs worldwide, underscoring the urgency of conservation strategies [3,4]. Cryopreservation, a method for preserving biological materials at low temperatures to ensure their vital functions and viability remain intact upon thawing [5,6,7], has been employed on a variety of substances, such as coral gametes and fish genetic material [8,9]. This technique holds promise for the conservation of endangered species and for facilitating advancements in developmental biology and marine biotechnology [10,11]. Despite its potential, cryopreservation poses formidable challenges. For example, ice crystals may form [12,13], which may result in damage to cells and tissues and increase the risk of contamination by pathogenic agents [14].Vitrification, an advanced cryopreservation method, rapidly cools biological samples to freezing temperatures in a glass-like medium, preventing ice crystal formation. Although vitrification is not yet widely established in aquatic cryopreservation, it has been experimentally applied to a limited number of invertebrate species and offers potential advantages over slow-cooling methods, including simplified equipment requirements and shorter processing times [8].

While sperm cryopreservation has achieved considerable success across various aquatic species, including corals, this is primarily accomplished through slow-freezing methods [8,9,15]. In contrast, the cryopreservation of oocytes and embryos remains challenging due to their complex membrane systems, high yolk and lipid content, and low permeability to solutes [16,17,52]. Vitrification is an ultra-rapid cooling technique that uses high concentrations of cryoprotective agents (CPAs) to prevent ice formation has been applied experimentally to some aquatic invertebrates [8], but it is not yet widely adopted. A key limitation is the cytotoxicity and osmotic stress caused by high CPA concentrations, which can lead to cellular damage, especially in large oocytes [18,19,20,21,22]. Despite these drawbacks, vitrification offers potential advantages over slow cooling, including reduced equipment needs, shorter processing times, and avoidance of ice crystal formation [11,7].

In coral cryopreservation, coral cells are subjected to low temperatures, which may cause membrane damage and diminished cryotolerance [23,24,25] due to thermotropic events that alter the physical properties of membrane lipids during freezing and thawing, causing the membranes to transition from a fluid liquid crystalline phase to a rigid gel phase [24,26]. These changes can result in cryoinjuries [24]. Studies have identified a direct correlation between the degree of cryoinjury and the membrane lipid phase transition in the oocytes of three gorgonian corals: *J. juncea*, *J. fragilis*, and *Ellisella robusta* [24]. Additionally, exposure to chilling can affect lipid behavior [27,24]. Studies have employed liposome supplementation to enhance coral larval cryotolerance and improve cryopreservation outcomes to address these challenges [27]. In addition, lipid profiling can be employed to cryobank coral propagules and ensure their development following thawing [28].

While previous studies have demonstrated the feasibility of vitrification for coral oocytes and the positive effects of lipid supplementation in coral larvae [24], the effects of specific lipid types on coral oocyte cryotolerance remain poorly understood. In particular, deep-sea gorgonian species, which have higher membrane lipid content, may respond differently to lipid-enriched vitrification protocols. Previous studies have not systematically evaluated the comparative effects of multiple lipid types incorporated into vitrification solutions (VSs). Therefore, this study investigates the influence of eight selected lipids on the post-thaw viability of *J. juncea* and *J. fragilis* oocytes. The results aim to identify optimal lipid additives to improve vitrification protocols and support long-term coral cryobanking and reef restoration efforts.

## Materials and methods

### Coral collection and oocyte isolation

*J. fragilis* and *J. juncea* specimens were collected during their reproductive season (June–September) from Kenting National Park, Nanwan, Taiwan (21°56’N, 120°44’E) at depths of 3–10 m. The National Kenting Park Management Office of Taiwan issued a coral collection permit to the study authors. Professional divers subsequently collected the permitted quantities of *J. fragilis* and *J. juncea* branches, which were then transported to the coral husbandry center of the National Museum of Marine Biology and Aquarium in Checheng, Taiwan. Upon arrival, the branches were affixed to substrates with bindings and placed in half-ton tanks with gravel bases. Native seawater with a salinity of 35 ppt was circulated through the tanks, which were maintained at a constant temperature of 25°C. Each branch measured approximately 60 cm and was analyzed and categorized by oocyte type and quantity before being transferred to the husbandry facility.

A scalpel was employed to cut a small incision in the coral coenenchyme tissue for the experiments involving the oocytes. *Junceela* coral has especially large quantities of oocytes, rendering it an ideal experimental subject [26]. Caution was exercised to ensure the incision was deep enough to reach the oocytes beneath the polyp without exposing the coral skeleton. The excised coenenchyme tissue was placed in a Petri dish containing native seawater filtered through 0.4-µm filter paper in preparation for oocyte isolation. Under a dissecting microscope (SZ51,Olympus, Tokyo, Japan), individual oocytes were carefully extracted from the polyp cavity using tweezers. Only oocytes with an intact spherical shape, clear cytoplasm, and no visible membrane damage were selected. To ensure consistency in developmental stage and cryotolerance, we included only oocytes with a diameter larger than 250 μm, as smaller or morphologically irregular oocytes may be immature or nonviable. Oocytes ≥250 µm represented approximately 60–70% of the total isolated oocyte population during the reproductive season. Selected oocytes were immediately transferred from the Petri dish to a smaller container with filtered seawater for further analysis. The oocytes were not fertilized in this study. Instead, oocyte viability was assessed based on Adenosine triphosphate (ATP) levels to evaluate the effects of vitrification and lipid supplementation. Assessing fertilization competence and post-thaw development will be the focus of future work.

### Effects of vitrification solution on oocytes

The concentrations of CPAs used in the production of Equilibration Solution 1 (ES1), ES2, and VS are presented in Table 1. These solutions sequentially prepared the oocytes for exposure to the VS. This experimental phase was used to evaluate the toxicity of ES1 (1 M propylene glycol (PG) + 0.25 M ethylene glycol (EG) + 0.5 M methanol), ES2 (2 M PG + 0.5M EG + 1 M methanol), and VS (4 M PG + 1 M EG + 2 M methanol) to the oocytes. In each experiment, 30–50 freshly harvested oocytes were immersed in one of the three solutions in a six-well culture dish. For ES1 and ES2, five oocytes were removed at three intervals: 5, 10, and 15 minutes. ATP counts were used to determine oocyte viability. Five oocytes were subjected to viability tests in VS at 2-, 4-, and 8-minute intervals. These experiments were conducted under two temperature conditions: room temperature (26°C) and an ice bath (5°C).

**Table 1:** Concentrations of CPAs in ES1, ES2, and VS.

CPA Solutions	Propylene glycol (PG)	Ethylene glycol (EG)	Methanol
ES1	1M	0.25M	0.5M
ES2	2M	0.5M	1M
VS	4M	1M	2M

### Incorporation of liposomes into VS for vitrification

To investigate the effect of lipid composition on oocyte cryotolerance, nine lipid types were selected based on their structural roles in cellular membranes and previously reported cryoprotective properties in gametes and embryos. The lipids included phospholipids (PLs: phosphatidylcholine (PC) and phosphatidylethanolamine (PE), monounsaturated fatty acids (MUFAs: erucic acid, oleic acid, palmitoleic acid), and polyunsaturated fatty acids (PUFAs: docosahexaenoic acid, linoleic acid, and eicosatrienoic acid).PLs such as PC and PE are integral to coral oocyte membranes and influence membrane integrity under low-temperature conditions. MUFAs were chosen for their ability to maintain membrane fluidity and reduce chilling injury, while PUFAs were included due to their reported benefits in vertebrate cryopreservation models, although their effects in marine invertebrates remain uncertain.

The concentrations of CPAs in the VS are presented in Table 2, namely, PC (Supelco, Bellefonte, PA, USA), PE (Supelco), erucic acid, palmitoleic acid, docosahexaenoic acid, oleic acid, linoleic acid, and eicosatrienoic acid (all from Supelco). A quantity of 10 mg, combined with 250 μL of a 2:1 chloroform/methanol solution, of each lipid, was placed in a vial and thoroughly mixed. Next, 10 μL of this lipid solution was added to 1 mL of VS1 and mixed vigorously with a Vortex Genie 2 (Scientific Industries, Bohemia, NY, USA) to achieve a final 400 μg/μL concentration. The control vitrification was performed using VS without additional lipids.

**Table 2:** VS with different combinations of CPA concentrations.

VS designed for *Junceella* oocytes				
CPA VS	Propylene glycol (PG)	Ethylene glycol (EG)	Methanol	Lipid
VS concentrations	4M	1M	2M	200μg/mL

In each experiment, a cryotop (Kitazato, Tokyo, Japan) was used as the carrier for approximately 1 μL of VS, which was rapidly immersed in liquid nitrogen to initiate vitrification. A solution was deemed vitrified when it transitioned to a glass-like, transparent state without ice crystal formation. By contrast, droplets that appeared opaque and formed visible ice crystals upon immersion in liquid nitrogen were considered frozen. Oocytes were thawed by transferring the cryotop from the liquid nitrogen into the original VS. The vitrification or devitrification of the VS droplets was recorded during the freezing and thawing stages.

### Vitrification

This study assessed the effects of stepwise vitrification on oocytes through sequential exposure to ES1, ES2, and VS at a temperature of 5°C. In the first phase, coral oocytes were immersed in the ES1 in a six-well culture dish for 5, 10, or 15 minutes (Table 3). At the conclusion of the specified time, the ES1 was removed from the oocytes by using a pipette and was replaced with ES2 for 5 or 10 minutes. Following treatment with ES2, the solution was substituted with VS for an additional 2 minutes. Subsequently, five oocytes were placed in separate 1-μL droplets of VS on a cryotop by using pipette suction. The cryotop with the oocytes was then submerged in liquid nitrogen for more than 10 minutes to achieve vitrification. After vitrification, the oocytes were thawed in VS2 (5°C) to prevent the formation of ice crystals, and their viability was evaluated using an ATP assay.

**Table 3:** Oocyte immersion times in ES1, ES2, and VS.

Trail levels	ES1	ES2	VS
		Time (Min)	
Trail 1	15	10	2
Trail 2	15	5	2
Trail 3	10	10	2
Trail 4	10	5	2
Trail 5	5	10	2
Trail 6	5	5	2

### Viability assay

Oocyte viability was evaluated by measuring ATP levels with an ApoSENSOR Cell Vitality Test Kit (BioVision, Cambridge BioScience, Cambridge, UK). ATP production in mitochondria reflects cellular health, with lower ATP levels indicating increased damage from treatment. A luminescence assay was used to assess oocyte vitality. In each assay, five oocytes were extracted from the VS, placed in a glass vial containing 100 μL of nucleotide-releasing buffer, and incubated for 3–5 minutes. Subsequently, 5 μL of ATP monitoring enzyme was added, and the mixture was allowed to settle and mix thoroughly for 30 s to 1 minute. The oocytes were then analyzed using a luminometer (Lumat LB 9507, Berthold Technologies, Bad Wildbad, Germany) to determine ATP levels.

### Statistics

Statistical analyses were performed using SPSS (version 17.0). Data were first tested for normality using the Kolmogorov–Smirnov test and for homogeneity of variance using Levene’s test (*P > 0.05*). When these assumptions were met, differences between treatments were assessed using one-way ANOVA with least significant difference post hoc testing. If assumptions were not met, Welch’s ANOVA with Games–Howell post hoc analysis was applied. ATP concentrations were normalized relative to baseline levels for each experimental run to account for inter-trial variability. All results are presented as means ± SEM, and significance was defined as *P < 0.05*.

## Results

### Analysis of properties of VS with incorporated lipids

Experiments revealed vitrification in all VS incorporating lipids (Table 4). The VS droplets retained their transparency upon immersion in liquid nitrogen, indicating successful vitrification.

**Table 4:** Properties of VS with various incorporated lipids.

Lipid types	Trial 1		Trial 2		Trial 3	
In LN2	In VS	In LN2	In VS	In LN2	In VS
PC	V	V	V	V	V	V
PE	V	V	V	V	V	V
Palmitoleic acid	V	V	V	V	V	V
Docosahexaenoic acid	V	V	V	V	V	V
Oleic acid	V	V	V	V	V	V
Linoleic acid	V	V	V	V	V	V
Linolenic acid	V	V	V	V	V	V
Erucic acid	V	V	V	V	V	V
Eicosatrienoic acid	V	V	V	V	V	V

V: Vitrified

Effects of stepwise vitrification (ES1, ES2, and VS) on oocyte viability

**Fig 1:**
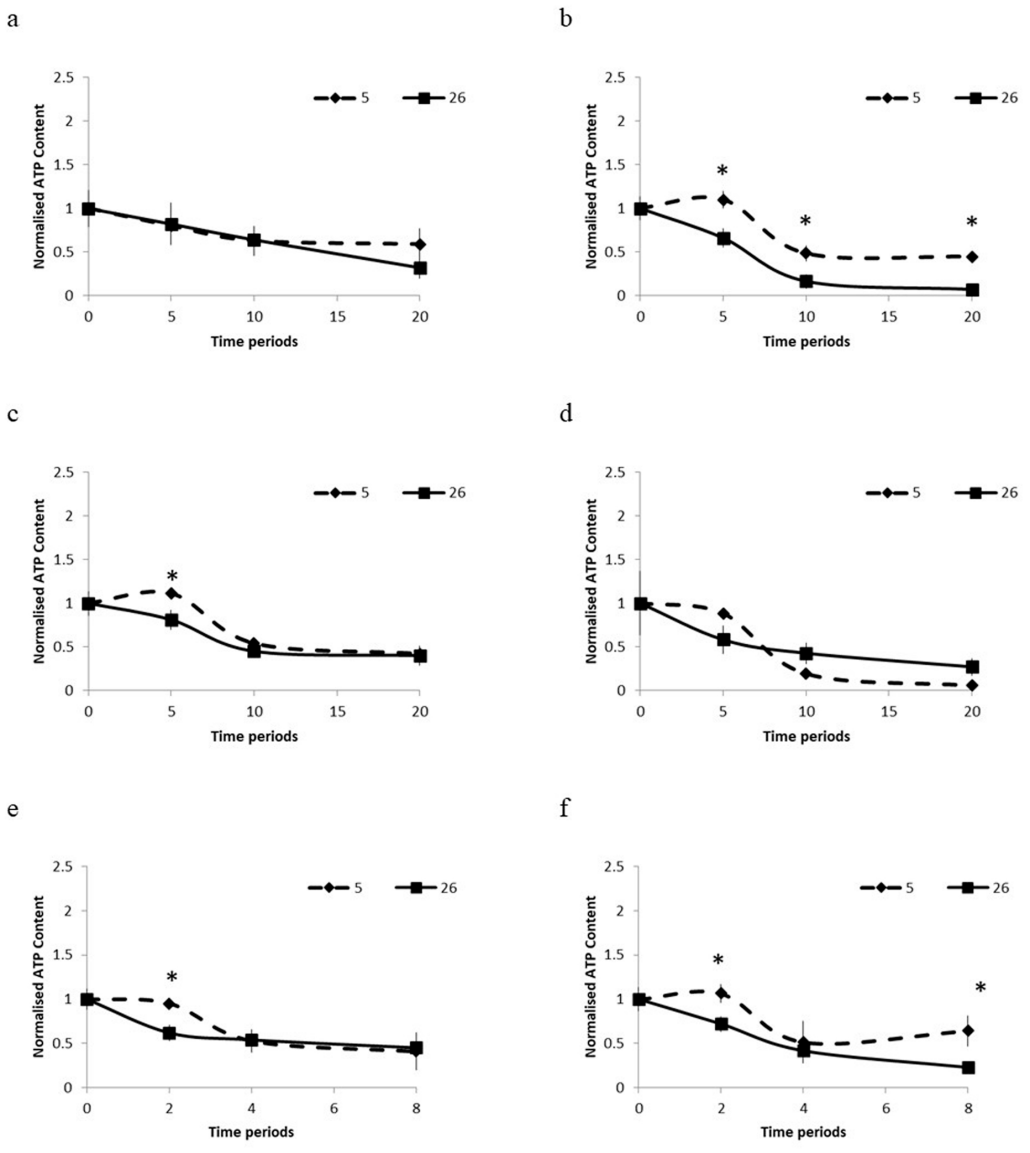
Effects of propylene-glycol-based ES1 (a,b), ES2 (c,d, and VS (e,f) on the *J. juncea* and *J. fragilis* oocytes at 5°C and 26°C over different time periods. Letters and numbers represent statistical differences between exposure time points for oocytes treated with the same solutions (*P < 0.05 difference between temperatures).

The oocytes of *J. juncea* and *J. fragilis* were immersed in ES1 solution for 20 minutes at 26°C and 5°C, as illustrated in Fig 1. Compared with higher concentrations of ES2 and VS2, low concentrations of ES1 had a diminished effect on the oocytes, rendering identification of clear trends in the viability of the *J. juncea* oocytes difficult (Fig. 1a). By contrast, the *J. fragilis* oocytes demonstrated a notable difference in viability, reflected in a difference between the initial and final ATP levels at both 26°C and 5°C (Fig. 1b), revealing a heightened sensitivity to ES2 compared with that of *J. juncea*, which exhibited more resilience at both temperatures (Fig. 1c). Immersion of the *J. juncea* oocytes in ES2 revealed no clear difference in ATP levels between the start and end of the experiments at 26°C and 5°C. Nevertheless, these oocytes exhibited significantly higher survival rates at 5°C than at 26°C after 5 minutes of exposure (*P* < 0.05; Fig. 1c), indicating enhanced resistance to vitrification at the lower temperature. This resistance was not observed in *J. fragilis* (Fig. 1d).

*J. juncea* oocytes exhibited greater cryotolerance than *J. fragilis* did when subjected to VS, particularly at 5°C (Fig. 1e, d). Both species exhibited superior viability at 5°C than at 26°C during VS storage (Fig. 1e, f). Although the initial ATP levels of *J. juncea* were higher (*P* < 0.05; Fig. 1e, f), *J. fragilis* exhibited unexpectedly greater viability after 8 minutes at low temperatures, indicating potential resistance to VS toxicity. Notably, the viability of both species was reduced in VS at 26°C (Fig. 1e, f), indicating an adverse reaction to the VS treatment. These results underscore the greater cryotolerance of *J. juncea* compared to *J. fragilis*, particularly under lower temperature conditions, and demonstrate the importance of temperature optimization in vitrification protocols.

### Incorporation of lipids for vitrification

**Fig 2:**
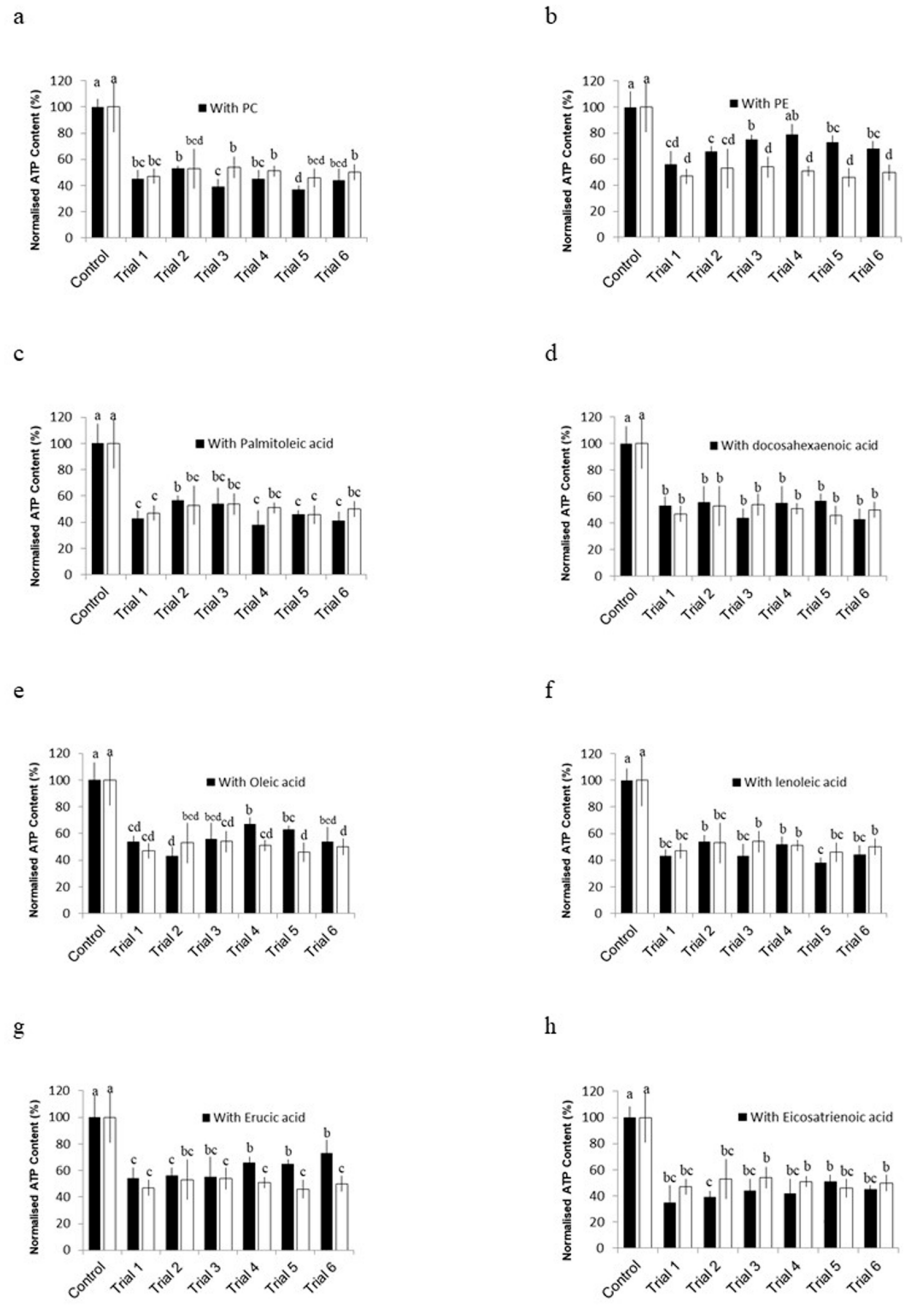
Normalized ATP concentrations in *J. juncea* oocytes after vitrification using equilibration solutions (ES) ES1, ES2, and VS incorporating (a) phosphatidylcholine (PC), (b) phosphatidylethanolamine (PE), erucic acid, (c) palmitoleic acid, (d) docosahexaenoic acid (e) oleic acid, (f) linoleic acid, (g) erucic acid, and (h) eicosatrienoic acid. Data are presented as mean ± SEM (n = 15). Letters above bars indicate statistically significant differences between groups within each panel (one-way ANOVA, *P < 0.05*).

**Fig 3:**
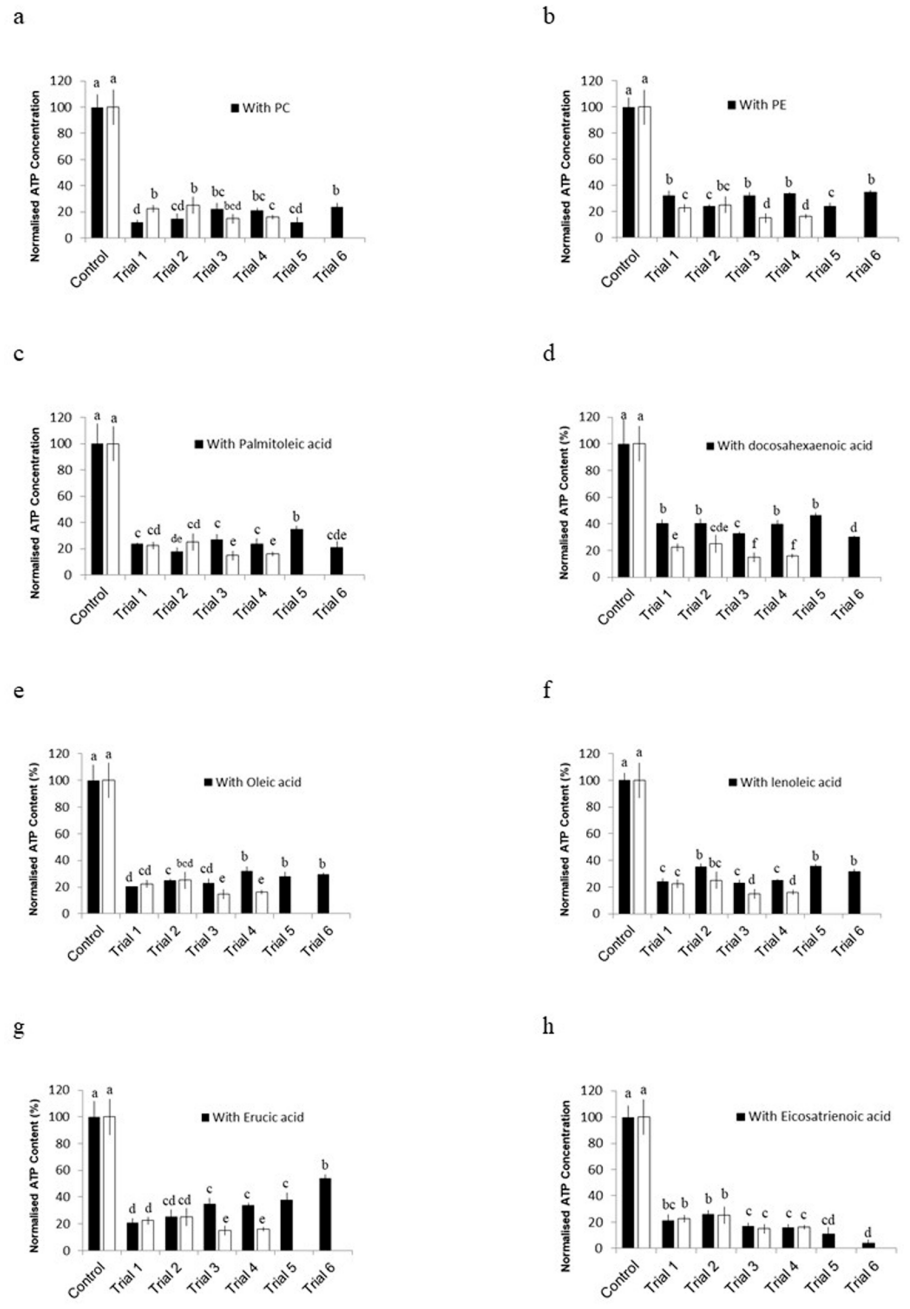
Normalized ATP concentration in *J. fragilis* oocytes after vitrification using ES1, ES2, and VS incorporating (a) PC, (b) PE, (c) palmitoleic acid, (d) docosahexaenoic acid, (e) oleic acid, (f) linoleic acid, (g) erucic acid, and (h) eicosatrienoic acid. Letters represent statistical differences between exposure time points for oocytes treated under the same conditions (*P* < 0.05). Data are presented as mean ± SEM (n = 15). Letters above bars indicate statistically significant differences between treatment groups within each panel (one-way ANOVA, *P < 0.05*).

The effects of liposome-incorporated VS on the vitrification process of the *J. juncea* and *J. fragilis* oocytes are illustrated in Figs 2 and 3 and presented in Table 5. In *J. juncea* (Fig 2), PC consistently reduced oocyte viability across trials, particularly in trial 3 compared with that in the oocytes treated with VS that did not contain PC (Fig 2a). This trend was also observed in the *J. fragilis* oocytes. In contrast, PE significantly increased viability in trials 3–6, with the highest viability observed in trial 4 (78.92% ± 7.86%; Fig 2b). No significant difference in viability was observed when palmitoleic acid, docosahexaenoic acid, or linoleic acid was incorporated in any trial (Figs 2c, d, f). The viability of *J. juncea* oocytes significantly improved in trials 4 and 5 when oleic acid was incorporated into VS, reaching a peak of 67.20% ± 5.11% in trial 4 (Fig 2e). Among the fatty acids tested, oleic acid and erucic acid both significantly enhanced viability in trials 4–6. Erucic acid produced the highest viability (73.12% ± 9.96%) in trial 6, indicating strong cryoprotective potential (Fig 2g). By contrast, eicosatrienoic acid-incorporated VS did not affect the viability of the *J. juncea* oocytes in any trial (Fig 2h), and similar results were obtained for palmitoleic acid, docosahexaenoic acid, and linoleic acid.

**Table 5:** Viability of *J. juncea* and *J. fragilis* oocytes after vitrification in the three most beneficial VSs.

Species	*Junceella Juncea*			*Junceella fragilis*		
**Lipid**	*Erucic acid*	*Oleic acid*	*PE*	*Erucic acid*	*Oleic acid*	*Docosahexaenoic acid*
**Trial**	Trial 6	Trial 4	Trial 4	Trial 6	Trial 5	Trial 5
**Viability**	73.12 ± 9.96	67.20 ± 5.11	78.92 ± 7.86	54.12 ± 2.99	35.78 ± 1.27	46.46 ± 1.75

In *J. fragilis* (Fig 3), PC also decreased viability in trials 1 and 2 (Fig 3a), while PE and palmitoleic acid improved viability in trials 3 and 4 (Figs 3b, c). Notably, the *J. fragilis* oocytes exhibited a significant improvement in viability when treated with docosahexaenoic acid-incorporated VS in all trials compared with that of specimens treated with VS that did not incorporate this liposome (Fig 3d). Oleic acid and linoleic acid both improved viability in trials 3 and 4 (Figs 3e–g), and erucic acid again yielded the highest viability in trial 6 (54.12% ± 2.99%; Fig 3g). In contrast, eicosatrienoic acid was associated with the lowest viability levels in *J. fragilis*, especially in trials 3–6 (Fig 3h). The *J. juncea* oocytes exhibited the highest viability (78.92% ± 7.86%) when they were treated with PE-incorporated VS in trial 4, followed by when they were treated with erucic acid (73.12% ± 9.96%) in trial 6 and with oleic acid (67.20% ± 5.11%) in trial 4 (Table 5). By contrast, PE incorporation did not significantly improve *J. fragilis* oocyte viability. For the *J. fragilis* oocytes, the highest viability (54.12% ± 2.99%) was observed in those treated with erucic acid in trial 6, followed by in those treated with docosahexaenoic acid (46.46% ± 1.75%) and oleic acid (35.78% ± 1.27%) in trial 5 (Table 5). Notably, incorporation of erucic acid resulted in high viability for both *J. juncea* and *J. fragilis* oocytes.

**Fig 4:**
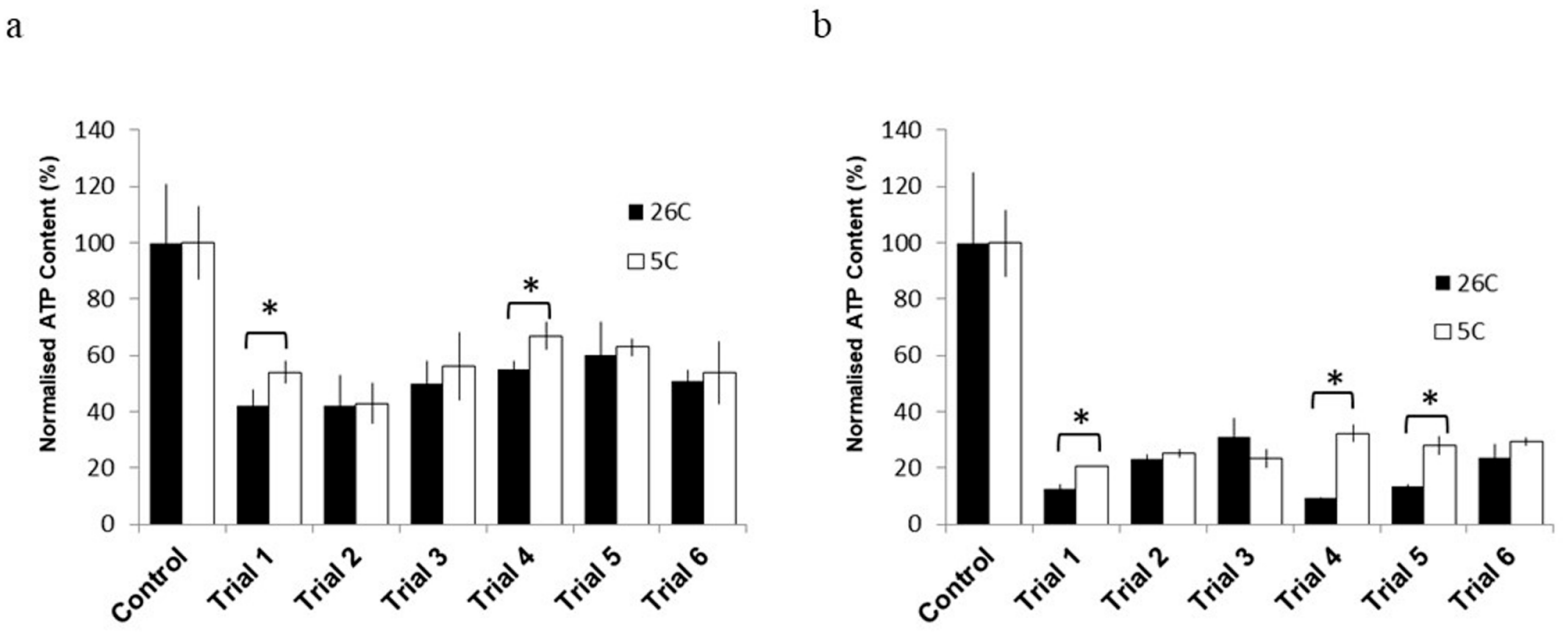
Normalized viability of vitrified *J. juncea* (a) and *J. fragilis* (b) oocytes with oleic‑acid–incorporated VS at 5 °C and 26 °C across six trials. Data are presented as mean ± SEM (n = 15 oocytes per point). An asterisk (*) indicates a significant difference between the two temperatures within the same trial (Welch’s ANOVA with Games–Howell post hoc test, *P < 0.05*).

The results of a comparison of oocyte viability at 5°C and 26°C between *J. juncea* and *J. fragilis* following vitrification with oleic-acid-incorporated VS are illustrated in Fig 4. Both species exhibited substantially improved oocyte viability after vitrification with oleic-acid-incorporated VS following equilibration at 5°C (Fig. 4). The oocytes in trials 1–4 maintained substantially higher viability at 5°C than at 26°C (Fig. 4a, b). By contrast, in trial 5, only the *J. fragilis* oocytes demonstrated similar improvements (Fig. 4b).

Overall, the most significant finding was that erucic acid consistently yielded the highest post-vitrification oocyte viability in both *J. juncea* and *J. fragilis*. In *J. juncea*, PE supplementation resulted in the highest recorded viability (78.92% ± 7.86%), while in *J. fragilis*, docosahexaenoic acid and oleic acid also significantly improved viability. These results highlight the strong cryoprotective potential of erucic acid across species and the species-specific benefits of other lipids.

## Discussion

CPAs are essential to the success of vitrification protocols because they mitigate ice formation during the cooling process [29]. PG, EG, and methanol are excellent CPAs for vitrification because of their low cytotoxicity and rapid permeability across cellular membranes [11,30]. These characteristics are crucial for cooling oocytes, promoting the maintenance of cell concentration gradients, and preventing cellular shrinkage due to dehydration from ice formation [18,19,9,31]. In the current study, the VS comprised 4 M PG, 1 M EG, and 2 M methanol. These CPAs have demonstrated beneficial effects in the vitrification of *J. juncea* and *E. robusta* oocytes [26,32]. Even with minor adjustments in CPA concentrations (an increase in the concentration of PG and a decrease in the concentration of EG), the VS maintained its vitrifying efficacy.

Study reported that direct exposure of oocytes to high-concentration VS can result in cryoinjuries [33,34,35,29,36]. Consequently, gradually employing low-concentration ES is essential to minimize cellular stress [26]. Sequentially introducing CPAs is vital for enhancing the post-cryopreservation viability of gorgonian coral oocytes. The process involves immersing the oocytes in ES containing low CPA concentrations and gradually transitioning them to VS with higher CPA levels. This approach mitigates ice crystallization damage during the freeze–thaw cycle [26] and reduces the risk of osmotic stress in oocytes, which may adversely affect their survival [20]. This technique has proven effective in increasing the survival rates of rat embryos following cryopreservation [37,38].

In the present study, the viability of oocytes immersed in various solutions was influenced by temperature. The viability of the oocytes remained comparable at 5°C and 26°C when they were exposed to ES1 but considerably declined when they were exposed to VS. The observed higher tolerance of the *J. juncea* oocytes to 5°C during exposure is consistent with the results of previous studies, in which minimal adverse effects from VS treatment at 5°C relative to those at 26°C were reported [26]. Although camel oocytes maintain some metabolic activity at 26°C despite the toxic conditions, lower temperatures generally enhance oocyte preservation across species. Studies on bovine embryos have demonstrated that cooler temperatures [39] and reduced enzymatic activity and CPA-related damage [40,9,41,42] contribute to this viability.

Integrating CPAs with additional components, such as lipids can improve cryopreservation outcomes by stabilizing cell membranes [43,44]. Previous studies have shown that external lipid supplementation enhances the freezing tolerance of coral larvae and promotes successful post-thaw development [27]. The present study extends the literature on cryopreservation by investigating the effects of lipid incorporation on the viability of oocytes in *J. juncea* and *J. fragilis* during vitrification. Although previous studies have reported beneficial effects of incorporating specific PUFAs, such as docosahexaenoic acid, linolenic acid, and eicosatrienoic acid, on embryo survival in cryopreservation [45,46], the findings of the present study reveal that have a detrimental effect on coral oocytes. This disparity in results may stem from the increased susceptibility of PUFAs to peroxidation because of their multiple double bonds [47].

At the molecular level, lipid composition influences cryo-tolerance by modulating membrane fluidity and oxidative stability. MUFAs like erucic and oleic acid help maintain membrane fluidity at low temperatures due to their kinked acyl chains, which disrupt lipid packing and reduce phase transitions that lead to cryo-injury [48]. In contrast, PUFAs such as docosahexaenoic acid is highly susceptible to lipid peroxidation because of their multiple double bonds, which may increase oxidative stress and impair membrane integrity [47,46]. PE, with its conical molecular structure, promotes membrane curvature and elasticity under cold stress, potentially explaining its protective effect on *J. juncea* oocytes during vitrification [25,49].

Studies have reported that boar semen samples frozen in a medium enriched with egg yolks containing high levels of MUFAs exhibited superior integrity to that of those preserved in a medium with high concentrations of saturated fatty acids. In gilthead seabream sperm, the flagellar membrane, which is rich in MUFA, demonstrated increased resistance to cold temperatures when treated with antifreeze proteins, whereas the head plasma membrane, which contains high levels of saturated fatty acids, exhibited less tolerance to chilling during cryopreservation [50]. However, a negative correlation between MUFA levels and human sperm viability following thawing, indicating that oleic acid may be prone to lipid oxidation, potentially reducing sperm motility [51]. The present study revealed that incorporating erucic acid and oleic acid into VS substantially improved the viability of the *J. juncea* and *J. fragilis* oocytes post-vitrification. Erucic acid was particularly effective in enhancing the viability of the oocytes from both *J. juncea* and *J. fragilis* following vitrification. This improvement may be due to the addition of MUFAs, which resulted in reduced synthesis of reactive oxygen species and increased ATP production, facilitating hyperactivation [52,53]. Consequently, these findings reveal erucic acid to be a promising agent for enhancing oocyte viability in *J. juncea* and *J. fragilis* during cryopreservation.

High Concentrations of PLs are widely employed to preserve the fluidity and permeability of the cell membrane by reducing its rigidity at low temperatures [25]. These substances are critical to stabilizing osmotic variations across the cell membrane [54]. Moreover, the oocytes of gorgonian corals such as *J. juncea* and *J. fragilis* contain greater amounts of PC and PE than do those of shallow-water corals because of a physiological adaptation to the colder temperatures and higher pressures in deeper ocean regions [25,24]. The present study demonstrated that the oocytes of *J. juncea* exhibited the highest viability when preserved with VS supplemented with PE. Notably, *J. fragilis* exhibited a general decline in cryotolerance after vitrification, irrespective of the type of liposome used. These results indicate that including PLs in VS can mitigate membrane alterations due to cold temperatures and thereby enhance oocyte viability following cryopreservation. The superior efficacy of PE may be due to its conical molecular structure, which improves membrane fluidity at low temperatures [49].

The present study assessed the effects of incorporating specific lipids into VSs on the oocytes of *J. juncea* and *J. fragilis*. While PE significantly improved the viability of *J. juncea* oocytes, it had no such effect on *J. fragilis*. In contrast, erucic acid markedly enhanced cryo-tolerance in both species, resulting in the highest post-vitrification viability. These findings highlight the importance of lipid selection in optimizing cryopreservation protocols. Further studies are needed to elucidate the mechanisms through which erucic acid confers its protective effects. By identifying lipid types that enhance cryo-tolerance especially erucic acid and PE, this study lays the groundwork for large-scale cryobanking of coral oocytes. Such optimized protocols can be used to establish genetic repositories for at-risk coral species, supporting assisted reproduction or reef repopulation following bleaching events [23,28]. In practice, this strategy supports long-term biodiversity conservation and can be integrated with larval rearing and outplanting programs to accelerate reef restoration under climate stress.

## Supporting information

S1 File Supporting info. (PDF)
