# Supplementary material for: Incorporation of lipids improves cryo-tolerance of vitrified gorgonian coral oocytes
Source: PLoS One. 2026 Feb 19;21(2):e0341229. doi: 10.1371/journal.pone.0341229 (PMC12919820; doi:10.1371/journal.pone.0341229)
Supplement: S1 File — (PDF) [file pone.0341229.s001.pdf]

W E 5C

439,635	479,352	319,844		412944	83036	47941
67,777	76,930	113,639		86115	24272	14013
88,737	80,857	147,237		105610	36264	20937
177,564	136,164	120,239		144656	29591	17084
141,904	129,343	152,295		141181	11493	6636
172,609	183,151	112,394		156051	38174	22040
216,459	247,516	206,383		223453	21440	12378

W L 5C

509,062	476,359	569,886		518436	47463	27403
138,403	135,628	106,767		126933	17519	10115
187,147	199,192	163,573		183304	18118	10460
113,549	145,874	100,324		119916	23433	13529
136,508	128,876	128,436		131273	4539	2620
176,883	198,389	181,155		185476	11385	6573
148,325	159,057	185,245		164209	18992	10965

W O 5C

522,291	415,645	349,753		429230	87067	50268
88,252	87,805	88,228		88095	251	145
118,276	107,210	98,701		108062	9815	5667
127,590	83,036	88,529		99718	24293	14026
154,569	113,340	147,530		138480	22054	12733
144,266	96,449	118,339		119685	23937	13820
121,453	136,895	121,112		126487	9015	5205

W D 5C

302,476	232,280	439,792		324849	105550	60939
124,719	148,483	122,720		131974	14332	8275
110,449	133,022	148,991		130821	19365	11180
103,925	103,243	114,225		107131	6153	3552
127,275	147,427	112,488		129063	17538	10126
141,497	150,150	161,162		150936	9856	5690
97,544	101,993	95,430		98322	3350	1934

W D 25

670,449	711,233	278,783		553488	238774	137856
66,983	87,806	51,373		68721	18279	10553
143,134	130,221	113,643		128999	14783	8535
246,052	143,178	123,344		170858	65871	38030
53,329	47,652	53,986		51656	3483	2011
67,303	82,827	73,202		74444	7836	4524
117,525	91,538	183,887		130983	47623	27495

R C

396990	412944	428898	412944	15954	9211
190234	194956	199678	194956	4722	2726
205723	217990	230258	217990	12267	7082
217140	223885	230629	223885	6744	3894
208063	211560	215058	211560	3498	2019
185681	191308	196935	191308	5627	3249
201902	207064	212226	207064	5162	2980

R E 5C

439,635	479,352	319,844	412,944	83,036	47,941
67,777	76,930	113,639	86,115	24,272	14,013
88,737	80,857	147,237	105,610	36,264	20,937
177,564	136,164	120,239	144,656	29,591	17,084
141,904	129,343	152,295	141,181	11,493	6,636
172,609	183,151	112,394	156,051	38,174	22,040
216,459	247,516	206,383	223,453	21,440	12,378

R L 5C

439,635	479,352	319,844	412,944	83,036	47,941
67,777	76,930	113,639	86,115	24,272	14,013
88,737	80,857	147,237	105,610	36,264	20,937
177,564	136,164	120,239	144,656	29,591	17,084
141,904	129,343	152,295	141,181	11,493	6,636
172,609	183,151	112,394	156,051	38,174	22,040
216,459	247,516	206,383	223,453	21,440	12,378

R O 5C

439,635	479,352	319,844	412,944	83,036	47,941
67,777	76,930	113,639	86,115	24,272	14,013
88,737	80,857	147,237	105,610	36,264	20,937
177,564	136,164	120,239	144,656	29,591	17,084
141,904	129,343	152,295	141,181	11,493	6,636
172,609	183,151	112,394	156,051	38,174	22,040
216,459	247,516	206,383	223,453	21,440	12,378

R D 5C

400035	399986	399988	400003	27.73	16.01
213136	213161	213121	213139.3	20.21	11.67
222752	222699	222722	222724.3	26.58	15.34
175778	175800	175755	175777.7	22.5	12.99
219590	219588	219622	219600	19.08	11.02
227932	227941	227934	227935.7	4.73	2.73
172956	172964	172939	172953	12.77	7.37

R O 25

395314	412944	430574	412944	17630	10179
169571	174772	179973	174772	5201	3003
163906	172936	181966	172936	9030	5213
200692	207442	214192	207442	6750	3897
223633	226225	228817	226225	2592	1496
239441	249138	258836	249138	9697	5599
206511	210103	213695	210103	3592	2074

W C 5C

402003	412944	423885	412944	10941	6317
90545	92887	95230	92887	2343	1353
98480	103790	109100	103790	5310	3066
58756	61685	64614	61685	2929	1691
65378	66693	68008	66693	1315	759

W PC 5C

404796	412944	421092	412944	8148	4704
48300	50063	51825	50063	1762	1017

57499	61018	64536	61018	3518	2031
84916	89381	93846	89381	4465	2578
86218	88063	89908	88063	1845	1065
44917	48414	51910	48414	3497	2019
94689	97317	99944	97317	2628	1517

W PE 5C

406955	412944	418933	412944	5989	3458
128122	131619	135116	131619	3497	2019
96902	98121	99339	98121	1218	703
129008	131260	133512	131260	2252	1300
140522	141279	142036	141279	757	437
96245	98257	100269	98257	2012	1162
143887	145039	146192	145039	1152	665

W P 5C

400220	412944	425668	412944	12724	7346
97717	98150	98583	98150	433	250
72670	75254	77838	75254	2584	1492
107522	110939	114355	110939	3417	1973
94338	97783	101227	97783	3444	1989
141117	143206	145295	143206	2089	1206
81995	85832	89670	85832	3837	2216

W E 5C

405784	412944	420104	412944	7160	4134
83728	88059	92390	88059	4331	2501
103680	106346	109013	106346	2667	1540
68067	70069	72072	70069	2003	1156
64687	66659	68631	66659	1972	1139
42506	46760	51015	46760	4255	2456
15906	17889	19873	17889	1984	1145

392262	412944	433626	412944	20682	11941
49688	51271	52854	51271	1583	914
94963	96243	97524	96243	1280	739

121767	127473	133178	127473	5705	3294
38237	38539	38841	38539	302	174
54862	55541	56220	55541	679	392
93599	97723	101848	97723	4125	2382

R PC 5C

407686	412944	418202	412944	5258	3036
178031	184038	190046	184038	6007	3468
215938	217874	219810	217874	1936	1118
157214	162480	167746	162480	5266	3040
178005	183993	189981	183993	5988	3457
149611	152196	154781	152196	2585	1492
173599	181251	188902	181251	7652	4418

R PE 5C

402877	412944	423011	412944	10067	5812
223827	232226	240625	232226	8399	4849
268159	271603	275047	271603	3444	1988
305050	308732	312414	308732	3682	2126
321932	328936	335941	328936	7005	4044
297724	301976	306229	301976	4252	2455
275016	280357	285699	280357	5341	3084

R P 5C

400774	412944	425114	412944	12170	7026
173826	178911	183995	178911	5085	2936
232079	234938	237797	234938	2859	1651
211374	221690	232006	221690	10316	5956
146166	155493	164820	155493	9327	5385
186264	188948	191632	188948	2684	1550
164925	170733	176541	170733	5808	3353

R E 5C

405776	412944	420112	412944	7168	4139
132645	143182	153718	143182	10537	6083
157598	162021	166444	162021	4423	2554
173432	181102	188772	181102	7670	4428

163198	172138	181077	172138	8939	5161
208089	212351	216613	212351	4262	2461
181810	184485	187159	184485	2675	1544

392262	412944	433626	412944	20682	11941
49688	51271	52854	51271	1583	914
94963	96243	97524	96243	1280	739
121767	127473	133178	127473	5705	3294
38237	38539	38841	38539	302	174
54862	55541	56220	55541	679	392
93599	97723	101848	97723	4125	2382

ES1

569320	370707	569115	503047	114610	66170	1.00	0.13
548543	472896	638454	553298	82881	47852	1.09	0.09
245616	320921	163620	243386	78674	45423	0.48	0.09
279150	208029	184996	224058	49081	28337	0.44	0.05
26C							
26							
514343	406634	504115	475031	59454	42040	1.00	0.08
232504	349555	359260	313773	70548	49885	0.66	0.10
51518	42739	140478	78245	54073.81	38236	0.16	0.08
42416	16621	41740	33592	14701.49	10396	0.07	0.02

ES1

0	569320	370707	569115	503047	114610	66170	1	0.13
5	548543	472896	638454	553298	82881	47852	0.8	0.12
10	245616	320921	163620	243386	78674	45423	0.63	0.17
20	279150	208029	184996	224058	49081.04	28337	0.59	0.18
	26C							
	26							
0	514343	406634	504115	475031	59453.6	42040	1	0.21
5	232504	349555	359260	313773	70548.1	49885	0.82	0.24
10	51518	42739	140478	78245	54073.81	38236	0.64	0.07
20	42416	16621	41740	33592	14701.49	10396	0.32	0.12

ES2

0	569320	370707	569115	503047	114610	66170	1	0.13
5	409729	450986	471101	443939	31287	18064	0.88	0.03
10	123935	72504	97216	97885	25722	14851	0.19	0.03
20	15345	35650	35451	28815	11666	6735	0.06	0.01
	26							
	26							
Control	198348	82285	91632	124088	64480	45594	1	0.37
5	52082	104752	59914	72249	28419	20095	0.6	0.16
10	35847	53474	69949	53090	17054	12059	0.43	0.12
20	42416	16621	41740	33592	14701.49	10396	0.27	0.1

ES2

569320	370707	569115	503047	114610	66170	1.00	0.13
548543	472896	638454	553298	82881	47852	1.09	0.09
245616	320921	163620	243386	78674	45423	0.48	0.09
279150	208029	184996	224058	49081	28337	0.44	0.05
26C							
26							
514343	406634	504115	475031	59454	42040	1.00	0.08
232504	349555	359260	313773	70548	49885	0.66	0.10
51518	42739	140478	78245	54073.81	38236	0.16	0.08
42416	16621	41740	33592	14701.49	10396	0.07	0.02

VS1

0	569320	370707	569115	503047	114610	66170	1	0.13
2	457123	513055	638647	536275	92963	53672	1.07	0.11
4	187998	494636	94674	259103	209247	120809	0.52	0.24
8	212939	495096	261521	323185	150848	87092	0.64	0.17
	26oC							
	26							
Control	146859	164729	131573	147720	16595	11734	1	0.08
5	127053	97419	94674	106382	17954	12695	0.72	0.09
10	49621	54194	80900	61572	16894	11946	0.41	0.08
20	42416	16621	41740	33592	14701	10396	0.23	0.07

VS1

569320	370707	569115	503047	114610	66170	1	0.09
457123	513055	638647	536275	92963	53672	0.95	0.06
187998	494636	94674	259103	209247	120809	0.53	0.13
212939	495096	261521	323185	150848	87092	0.41	0.21
26oC							
26							
146859	164729	131573	147720	16595	11734	1	0.12
127053	97419	94674	106382	17954	12695	0.62	0.09
49621	54194	80900	61572	16894	11946	0.54	0.04
42416	16621	41740	33592	14701.49	10396	0.45	0.09
